# A Nondestructive Measurement Method of Optical Fiber Young’s Modulus Based on OFDR

**DOI:** 10.3390/s22041450

**Published:** 2022-02-14

**Authors:** Pengfei Li, Cailing Fu, Huajian Zhong, Bin Du, Kuikui Guo, Yanjie Meng, Chao Du, Jun He, Lei Wang, Yiping Wang

**Affiliations:** 1 Key Laboratory of Optoelectronic Devices and Systems of Ministry of Education/Guangdong Province, College of Physics and Optoelectronic Engineering, Shenzhen University, Shenzhen 518060, China; lipengfei2022@163.com (P.L.); 2060453029@email.szu.edu.cn (H.Z.); dubean@126.com (B.D.); 2150190116@email.szu.edu.cn (K.G.); mengyanjie2020@email.szu.edu.cn (Y.M.); 2060453065@email.szu.edu.cn (C.D.); hejun07@szu.edu.cn (J.H.); ypwang@szu.edu.cn (Y.W.); 2 Shenzhen Key Laboratory of Photonic Devices and Sensing Systems for Internet of Tings, Guangdong and Hong Kong Joint Research Centre for Optical Fibre Sensors, Shenzhen University, Shenzhen 518060, China; 3 Shenzhen Key Laboratory of Polymer Science and Technology, College of Materials Science and Engineering, Shenzhen University, Shenzhen 518055, China; wl@szu.edu.cn

**Keywords:** optical frequency domain reflectometry, single mode fiber, Young’s modulus, nondestructive measurement

## Abstract

A nondestructive measurement method based on an Optical frequency domain reflectometry (OFDR) was demonstrated to achieve Young’s modulus of an optical fiber. Such a method can be used to measure, not only the averaged Young’s modulus within the measured fiber length, but also Young’s modulus distribution along the optical fiber axis. Moreover, the standard deviation of the measured Young’s modulus is calculated to analyze the measurement error. Young’s modulus distribution of the coated and uncoated single mode fiber (SMF) samples was successfully measured along the optical fiber axis. The average Young’s modulus of the coated and uncoated SMF samples was 13.75 ± 0.14, and 71.63 ± 0.43 Gpa, respectively, within the measured fiber length of 500 mm. The measured Young’s modulus distribution along the optical fiber axis could be used to analyze the damage degree of the fiber, which is very useful to nondestructively estimate the service life of optical fiber sensors immersed into smart engineer structures.

## 1. Introduction

Optical fiber Young’s modulus is an essential physics parameter in the field of optical fiber devices and their sensing applications. For example, Young’s modulus was used to study the shrinkage of the coating material in a quartz fiber [1], to analyze the micro bending losses induced by temperature drop in a double-coated fiber [2], and to predict the mechanical reliability of an optical fiber cable [3]. Various methods have been proposed to measure optical fiber Young’s modulus. As early as 2001, EI-Diasty et al. demonstrated a bent fiber Young’s modulus measurement method based on the multiple-beam interference technique with the help of the Fizeau fringes shifts [4]. In 2008, Antunes et al. reported a static load detection method for measuring Young’s modulus of a photosensitive and standard fiber [5]. In 2012, Sokkar et al. presented Young’s modulus of a polymeric fiber by use of a transverse interferometric method based on the phase distribution of the micro-interferograms [6]. In 2018, Huether et al. demonstrated an enhanced method to precisely determine Young’s modulus of single glass fibers by means of optical images to track the beads [7]. However, the optical fibers employed in [5,6,7] have to be cut off for measuring their Young’s modulus, which is a destructive behavior. Additionally, it is necessary to know the distributed Young’s modulus along the axis of an optical fiber in most practical applications to estimate the pressure sensitivity and analyze the bending state as a special optical fiber sensor (e.g., polymer optical fiber sensors) [8,9], but the aforementioned method can only measure Young’s modulus of one point on the optical fiber. So, it is significant to develop a nondestructive method for realizing a distributed measurement of optical fiber Young’s modulus. Optical frequency domain reflectometry (OFDR) is a spatially resolved method for analyzing optical backscattering in an optical fiber [10,11,12,13] and has been widely used to realize a distribution measurement of temperature [14,15], strain [16,17], shape [18,19], refractive index [20], and water level [21]. Nevertheless, the researchers have not focused on optical fiber Young’s modulus measurement based on an OFDR so far.

In this letter, we demonstrated a nondestructive measurement method based on an OFDR to achieve Young’s modulus of an optical fiber. Two types of SMF samples, i.e., the coated and uncoated SMFs, were employed to measure not only the averaged Young’s modulus within the measured fiber length, but also the Young’s modulus distribution along the optical fiber axis. Moreover, the standard deviation of the measured Young’s modulus is calculated to analyze the measurement error.

## 2. Test Design

**Figure 1:**
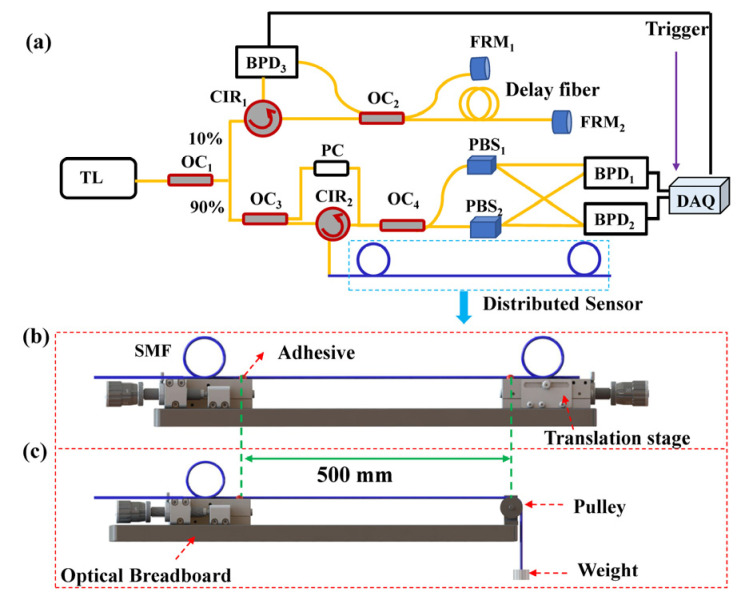
Experimental setup for measuring Young’s modulus of an optical fiber by use of (**a**) an optical frequency domain reflectometry (OFDR), in which a tensile strain is applied to the fiber by (**b**) a translation stage and (**c**) a weight, respectively. TL: tunable laser; OC: optical coupler; CIR: circulator; PC: polarization controller; PBS: polarization beam splitter; FRM: faraday rotating mirror; BPD: balanced photo-detector; DAQ: data acquisition card.

An experimental setup was designed and built to measure Young’s modulus of an optical fiber, as shown in Figure 1, the key components of the OFDR in Figure 1a include a tunable laser (TL), a polarization controller (PC), a data acquisition card (DAQ), two faraday rotating mirrors (FRM_1_, FRM_2_), two polarization beam splitters (PBS_1_, PBS_2_), three balanced photo-detectors (BPD_1_, BPD_2_, and BPD_3_), and four optical couplers (OC_1_, OC_2_, OC_3_, and OC_4_). The light from the TL is divided into two paths by OC_1_ (10/90). Ten percent of the light enters the auxiliary interferometer consisting of OC_1_, CIR_1_, OC_2_, FRM_1_, FRM_2_, and BPD_3_ as a trigger signal to monitor laser scan linearity. While 90% of the light enters the main interferometer consisting of OC_1_, OC_3_, CIR_2_, PC, OC_4_, PBS_1_, PBS_2_, BPD_1_, and BPD_2_ as a sensing signal to monitor the measured parameter change.

Furthermore, a measured optical fiber (blue line), is spliced to the pigtail of CIR2 in the OFDR. Two types of tensile strains are applied to the optical fiber employed to measure its Young’s modulus. As shown in Figure 1b, the two ends of the optical fiber employed are fixed on two translation stages, respectively, and then a tensile strain is applied to the optical fiber by moving the right translation stage. Alternatively, as shown in Figure 1c, one end of the optical fiber employed is fixed on a translation stage, and the other end of the optical fiber is connected with a weight through a pulley, and then a tensile strain is applied to the optical fiber by use of different weights.

**Figure 2:**
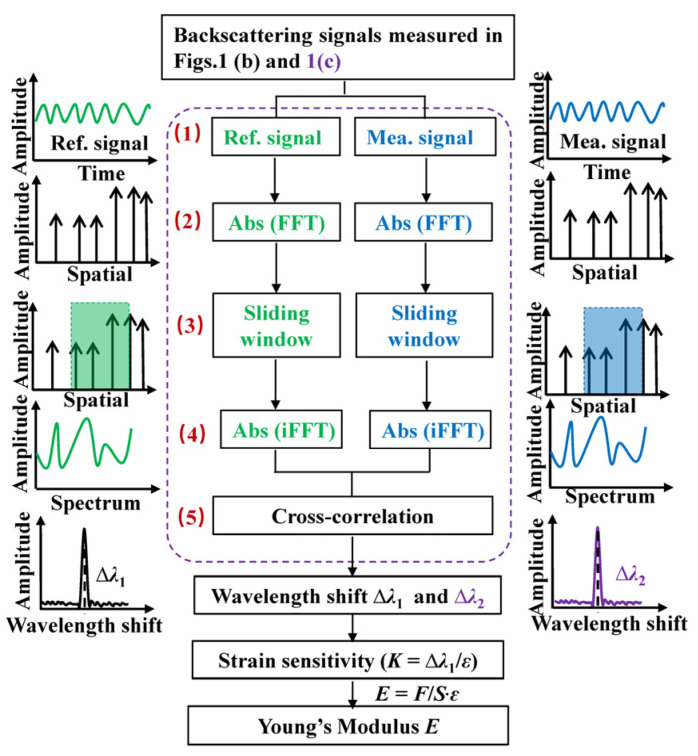
Signal demodulation process for calculating Young’s modulus through the backscattering signals measured in Figure 1. The Ref. and Mea. signals are illustrated by green and blue curves, respectively.

As reported in our previous works [22], the backscattering in SMF is induced by the inhomogeneous refractive index distributed in the core of the optical fiber, and the backscattering is stable once the SMF is fabricated. This indicated that Young’s modulus along the fiber could be obtained without damaging the fiber. Compared to methods in which the fiber needs to be to cut off, see [5,6,7], the OFDR method has incomparable advantages on measurement Young’s modulus of the optical fiber. In addition, Young’s modulus of the optical fiber employed can be achieved via the signal demodulation process illustrated in Figure 2. First of all, two backscattering signals in the time domain, i.e., the reference and measurement signals, are measured before or after a tensile strain is applied to the optical fiber, respectively. Secondly, the Ref. and Mea. signals are transformed from the time domain to the spatial domain by Fast Fourier Transform (FFT). Thirdly, the sliding window process is applied to the Ref. and Mea. signals in the spatial domain to obtain the local information in a segment of the optical fiber. Here, the width of the sliding window indicates the sensing spatial resolution. Fourthly, the local Ref. and Mea. signals in each of sliding windows are transformed from the spatial domain to the spectrum domain by Invert Transform (iFFT). Fifthly, the Ref. and Mea. signals in the spectrum domain are calculated by cross-correlation to obtain the backscattering spectrum wavelength shift (∆λ_1_) induced by the tensile strain in Figure 1b. Thus, the strain sensitivity of the optical fiber could be expressed as

(1) $$K=\frac{\Delta \lambda_{1}}{\varepsilon }=\frac{\Delta \lambda_{1}L}{\Delta L}$$

where *L* is the length of the measured fiber, ∆*L* is the moved distance of the right translation stage, namely the length change in the optical fiber stretched.

Then, the same optical fiber sample is stretched by a weight to measure its Young’s modulus, as shown in Figure 1c. Young’s modulus of the optical fiber can be calculated by

(2) $$E=\frac{F}{S\varepsilon }$$

where *F* is the tensile stress applied to the optical fiber, *S* is the cross-section aera of the optical fiber, and *ε* is the tensile strain applied to the optical fiber and can be calculated by

(3) $$\varepsilon =\Delta \lambda_{2}/K$$

where Δ*λ*_2_ is the strain-induced wavelength shift in the optical fiber while the weight is applied, as illustrated in Figure 1c, and can be calculated by the same process above in Figure 2. Thus, Young’s modulus (*E*) of the measured optical fiber can be expressed by

(4) $$E=\frac{4F}{\pi D^{2}}\frac{K}{\Delta \lambda_{2}}$$

where *D* is the diameter of the optical fiber.

## 3. Experimental Results and Discussions

In our experiments, two types of optical fiber samples were employed to measure their Young’s modulus. One sample, i.e., coated SMF, was a standard SMF (YOFC-G652), with a perfect polymer coat. Another sample, i.e., uncoated SMF, was the same type of SMF whose polymer coat was moved. As shown in Figure 1b, the tensile strain applied was increased from 0 to 1000 με with a step of 100 με to measure the strain-induced wavelength shift in the backscattering spectrum of the fiber. The wavelength shift (∆λ_1_) in the backscattering spectrum at each position, i.e., slide window, along the axis of the optical fiber was calculated by the process illustrated in Figure 2, where the sliding window width, i.e., the sensing spatial resolution, was 10.40 mm, as for a tuning range of 30 nm centered at 1550 nm and a number of 400 point in a sliding window.

**Figure 3:**
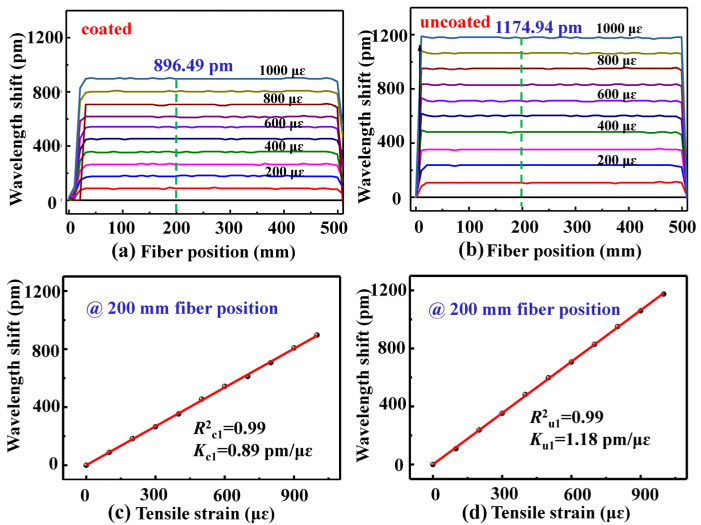
Wavelength shift evolution of the backscattering spectrum at the position from 0 to 500 mm along the axes of the (**a**) coated and (**b**) uncoated SMF samples while the tensile strain applied was increased from 0 to 1000 με with a step of 100 με. Wavelength shift in the backscattering spectrum at the position, e.g., 200 mm, in the (**c**) coated and (**d**) uncoated SMF samples as a function of the tensile strain applied.

**Figure 4:**
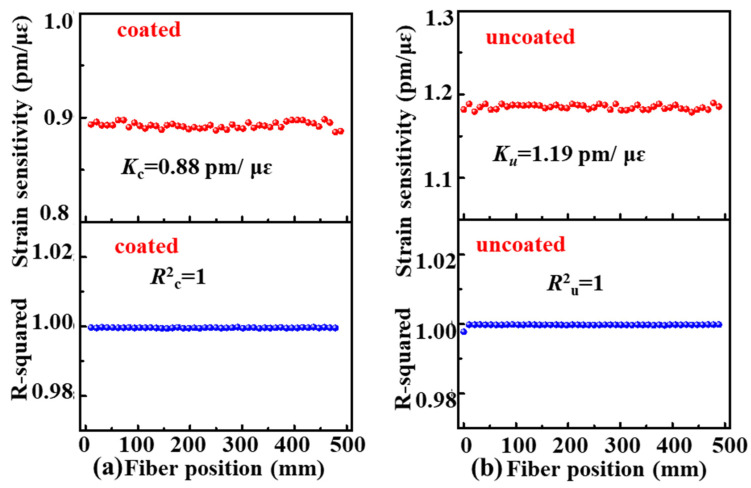
The calculated strain sensitivity and the R-Squared at each position from 0 to 500 mm along the axes of the (**a**) coated and (**b**) uncoated SMF samples.

As shown in Figure 3a,b, the backscattering spectrum wavelength in the coated and uncoated SMF samples shifted by about 896.49 and 1174.94 pm, respectively, while the tensile strain was increased to 1000 με. As shown in Figure 3c,d, the backscattering spectrum wavelength in the two SMF samples shifted linearly with a strain sensitivity of 0.89 and 1.18 pm/με, respectively, at the fiber position of 200 mm while the tensile strain increased from 0 to 1000 με. Moreover, as shown in Figure 4, the averaged strain sensitivity, i.e., *K_c_* and *K_u_*, of the backscattering spectrum wavelength shift at the position from 0 to 500 mm was about 0.88 and 1.19 pm/με along the axes of the coated and uncoated SMF samples, respectively. Moreover, the R-Squared, i.e., *R_c_* and *R_u_*, of the strain sensitivity coefficient was close to one at each position of the coated and uncoated SMF, which indicated that there was a very good linear relationship between the backscattering spectrum wavelength shift and the tensile strain applied.

**Figure 5:**
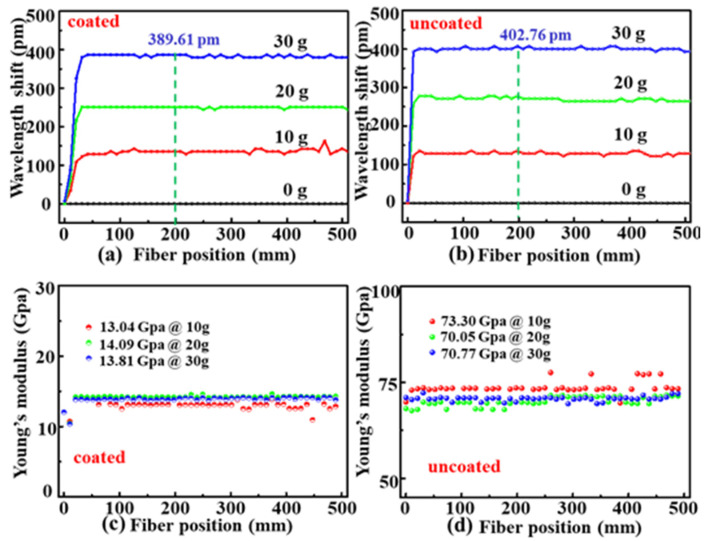
Wavelength shift evolution of the backscattering signal at the position from 0 to 500 mm along the axes of the (**a**) coated and (**b**) uncoated SMF samples while the weight applied was increased 10, 20, and 30 g. Calculated Young’s modulus distribution at each position of the (**c**) coated and (**d**) uncoated SMF samples.

In order to measure Young’s modulus of the coated and uncoated SMF samples, as shown in Figure 1c, a tensile stress, i.e., a weight of 10, 20, or 30 g, was gradually applied to the SMF samples. The strain-induced wavelength shift in the backscattering spectrum at each position along the fiber axis was calculated by the same process illustrated in Figure 2. As shown in Figure 5a,b, the backscattering spectrum wavelength in the coated and uncoated SMF samples shifted by about 389.61 and 402.76 pm, respectively, while the weight was increased to 30 g. Thus, according to Equation (4), Young’s modulus distribution at each position along the axes of the coated and uncoated SMF samples were calculated and illustrated in Figure 5c,d, respectively. Obviously, the measured value fluctuation is larger while the weight is 10 g than the weight is 20 and 30 g as the error of the Young’s modulus is inversely proportional to the change of wavelength shift. Therefore, a larger weight, e.g., 30 g, should be applied to measure precisely Young’s modulus of the optical fiber.

**Figure 6:**
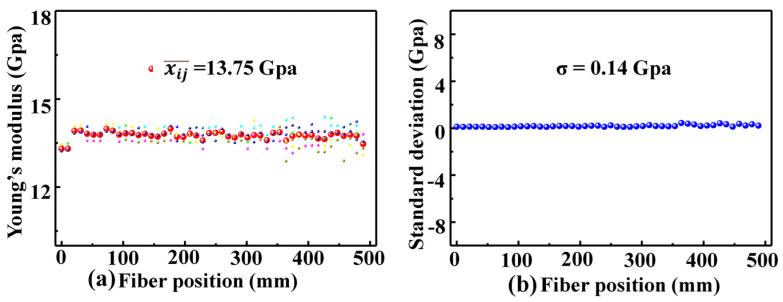
(**a**) Young’s modulus distributions for the measurements taken eight times and their average value distribution (●) in the coated SMF sample, (**b**) corresponding standard deviation distribution.

Analysis of the Young’s modulus measurement error in our OFDR system is illustrated in Figure 1; furthermore, a weight of 30 g was applied eight times to the coated SMF sample. Figure 6a illustrates the measured Young’s modulus distributions of the coated SMF sample. Moreover, as illustrated (●) in Figure 6a, the average value of the measured Young’s modulus for eight times at the *i*th position along the axis of the coated SMF sample can be expressed as

(5) $$\bar{x_{i}}=\frac{1}{8}\sum_{j=1}^{8}x_{ij}(i=1,2\ldots 8)$$

where *j* is the measurement times; *i* is the serial number of measurement position, i.e., slide windows, along the fiber axis. As shown in Figure 6b, the standard deviation distribution of the measured Young’s modulus at the ith position along the axis of the coated SMF sample can be expressed as

(6) $$\sigma_{i}=\sqrt{\frac{1}{8}\sum_{j=1}^{8}{\left(x_{ij}-\bar{x_{i}}\right)}^{2}}$$

Furthermore, the average value of the measured Young’s modulus of the coated SMF sample can be expressed as

(7) $$\bar{x_{ij}}=\frac{1}{N}\sum_{i=1}^{N}\left(\bar{x_{i}}\right)$$

where *N* is the number of the measured points, i.e., slide windows, along the optical fiber axis. *N* is 48 in our experiments. The Young’s modulus standard deviation, σ, of the coated SMF samples can be expressed as

(8) $$\sigma =\sqrt{\frac{1}{8}\sum_{i=1}^{N}\left(\bar{x_{i}}-\bar{x_{ij}}\right)}$$

According to Equations (5)–(8), the Young’s modulus average value of the coated SMF sample and its standard deviation are calculated to be 13.75 and 0.14 Gpa, respectively. Furthermore, the Young’s modulus of the uncoated SMF sample was also measured for eight times while a weight of 30 g was applied. The Young’s modulus average value of the uncoated SMF sample and its standard deviation are calculated to be 71.63 and 0.43 GPa, respectively. This indicates that the Young’s modulus average value of the coated and uncoated SMF agreed well with the result reported in [5,7].

**Figure 7:**
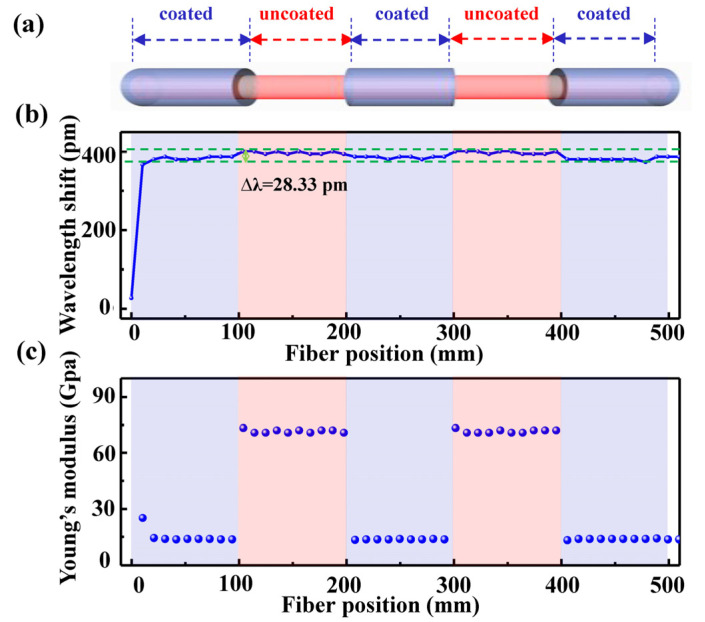
(**a**) Schematics diagram of the periodically coated and uncoated optical fiber, (**b**) Measured wavelength shift, and (**c**) Young’s modulus along the axis of the periodically coated and uncoated optical fiber.

The coated SMF with a length of 500 mm was placed along a scaled ruler and marked with markers at intervals of 100 mm. Then, the polymer coat was removed periodically along the mark with a wire stripper without damaging other coatings, as shown in Figure 7a; after which, the periodical coated and uncoated SMF sample was employed to measure its Young’s modulus distribution along the optical fiber axis by applying a weight of 30 g to the fiber end. As shown in Figure 7b, the strain-induced wavelength shift in the backscattering spectrum was periodically changed by about 28.33 pm along the optical fiber axis, which indicated the damage degree in the optical fiber sample. As a result, as shown in Figure 7c, Young’s modulus distribution was also periodically changed along the optical fiber axis, and Young’s modulus within the uncoated fiber section was higher than that within the coated fiber section. Obviously, the distributed measurement of Young’s modulus in the optical fiber was realized by use of the OFDR system illustrated in Figure 1.

## 4. Conclusions

In conclusion, a nondestructive measurement method based on an OFDR was successfully demonstrated to achieve Young’s modulus of the optical fiber. Such a method can be used to measure Young’s modulus distribution along the optical fiber axis. In contrast, traditional methods [4,5,6,7] can only be used to measure the averaged Young’s modulus within the measured fiber length. Moreover, no optical fiber was destroyed during our Young’s modulus measurements. Young’s modulus distribution of the coated and uncoated SMF samples were successfully achieved along the optical fiber axis with their average Young’s modulus being 13.75 ± 0.14 and 71.63 ± 0.43 Gpa, respectively, within the measured fiber length of 500 mm. The measured Young’s modulus distribution, corresponding to the wavelength shift distribution of the backscattering spectrum, along the optical fiber axis could be used to analyze not only the characteristics of the specialty optical fibers but also the damage degree in the fiber, which is very useful to nondestructively estimate the service life of optical fiber sensors immersed into the smart engineer structures and marine health structures.

## Data Availability

Not applicable.
